# Prospective assessment of risk biomarkers of sinusoidal obstruction syndrome after hematopoietic cell transplantation

**DOI:** 10.1172/jci.insight.168221

**Published:** 2023-05-22

**Authors:** Yan Han, Alan Bidgoli, Brittany P. DePriest, Alejandra Méndez, Khadijeh Bijangi-Vishehsaraei, Evelio D. Perez-Albuerne, Robert A. Krance, Jamie Renbarger, Jodi L. Skiles, Sung W. Choi, Hao Liu, Sophie Paczesny

**Affiliations:** 1 Department of Biostatistics and Health Data Science, Indiana University School of Medicine, Indianapolis, Indiana, USA.; 2 Department of Microbiology and Immunology, Medical University of South Carolina, Charleston, South Carolina, USA.; 3 Department of Pediatrics, Indiana University School of Medicine, Indianapolis, Indiana, USA.; 4 Department of Pediatrics, Children’s National Medical Center, Washington, DC, USA.; 5 Department of Pediatrics, Texas Children’s Hospital, and Baylor College of Medicine, Houston, Texas, USA.; 6 Department of Pediatrics, University of Michigan, Ann Arbor, Michigan, USA.; 7 Department of Biostatistics and Epidemiology, Rutgers School of Public Health, New Brunswick, New Jersey, USA.

**Keywords:** Oncology, Transplantation, Bone marrow transplantation

## Abstract

**BACKGROUND:**

Currently, no laboratory tests exist to stratify for the risk of developing sinusoidal obstruction syndrome (SOS), an early endothelial complication after hematopoietic cell transplantation (HCT). Risk biomarkers of SOS have not been verified in a prospective cohort accounting for differences between practices across institutions. Herein, we aimed to define risk groups for SOS occurrence using 3 proteins: L-ficolin, hyaluronic acid (HA), and stimulation 2 (ST2).

**METHODS:**

Between 2017 and 2021, we prospectively accrued 80 pediatric patients across 4 US centers. Biomarkers were tested by ELISA blind to patient groupings and associated with SOS incidence on day 35 after HCT, and overall survival (OS) on day 100 after HCT. Cutpoints were identified using retrospective cohorts and applied to the prospective cohort.

**RESULTS:**

Combination of the 3 biomarkers measured on day 3 after HCT in the prospective cohort provided 80% (95% CI 55%–100%) sensitivity and 73% (95% CI 62%–83%) specificity for risk of SOS occurrence. Patients with low L-ficolin were 9 times (95% CI 3–32) more likely to develop SOS, while patients with high HA and ST2 were 6.5 (95% CI 1.9–22.0) and 5.5 (95% CI 2.3–13.1) times more likely to develop SOS. These 3 markers also predicted worse day 100 OS — L-ficolin: HR, 10.0 (95% CI 2.2–45.1), *P* = 0.0002; HA: HR, 4.1 (95% CI 1.0–16.4), *P* = 0.031; and ST2: HR, 3.9 (95% CI 0.9–16.4), *P* = 0.04.

**CONCLUSION:**

L-ficolin, HA, and ST2 levels measured as early as 3 days after HCT improved risk stratification for SOS occurrence and OS and may guide risk-adapted preemptive therapy.

**TRIAL REGISTRATION:**

ClinicalTrials.gov NCT03132337.

**FUNDING:**

NIH.

## Graphical abstract

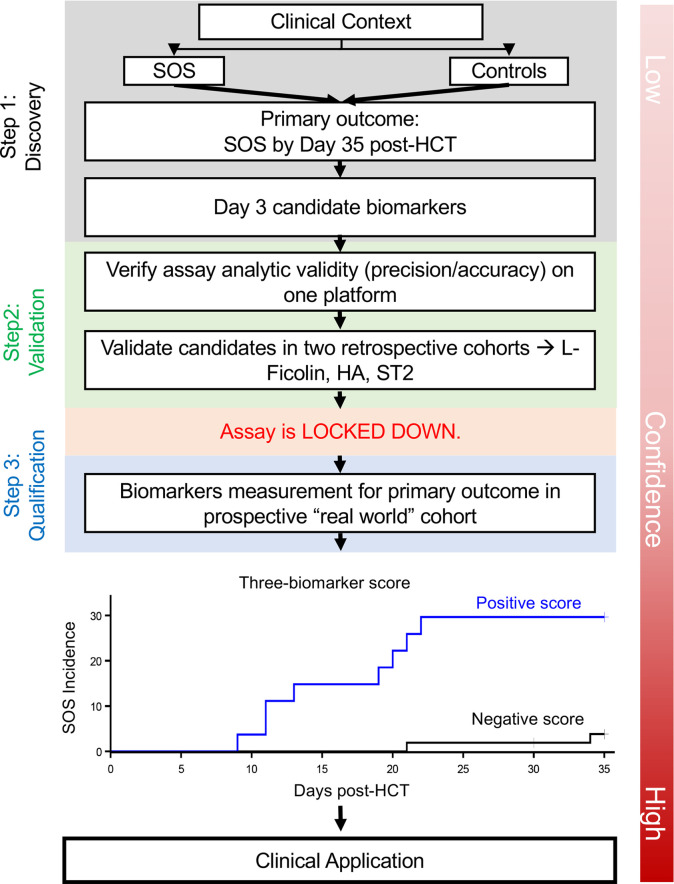

## Introduction

Allogeneic hematopoietic cell transplantation (HCT) is a potentially curative therapy for blood disorders. However, the efficacy of this procedure has been impeded by early endothelial dysfunction that can lead to a severe and potentially lethal complication called sinusoidal obstruction syndrome (SOS), also known as veno-occlusive disease (VOD) (1). Despite less aggressive conditioning regimens leading to a decrease in incidence in recent years, SOS that evolves to multiorgan failure (MOF) in children has a high mortality rate (2–4). As highlighted by the Pediatric Acute Lung Injury and Sepsis Investigators (PALISI) and Pediatric Blood and Marrow Transplantation Consortium (PTCTC) Joint Working Committee consensus, there is high variability in pediatric management of SOS, which may contribute to the increased morbidity and mortality (5).

Defibrotide has received Food and Drug Administration (FDA) approval for SOS treatment (6–8). The safety profile of defibrotide is excellent (9). In a randomized trial, its prophylactic administration in a pediatric population resulted in decreased SOS incidence, from 20% to 12%, although the *P* values were just at the limit of significance (*Z* test for competing risk analysis *P* = 0.0488; log-rank test *P* = 0.0507) (10, 11). This was not confirmed in a trial that included adults and children (10, 11). Therefore, prophylactic defibrotide for SOS is controversial and its administration to all HCT patients is not practical or cost efficient. To fill this gap, enrichment for patients at high risk of developing SOS is needed.

SOS diagnosis and its severity are assessed late in the course of the disease by nonspecific clinical signs and laboratory assays (ascites, weight gain, hepatomegaly, right upper quadrant pain, and bilirubin ≥ 2 mg/dL) (12–14). Updated scoring criteria have been proposed that allow for earlier recognition of SOS but came after the start of this prospective study (15, 16).

Currently, no validated laboratory test exists to stratify patients at high risk for developing SOS. Neither pretransplant clinical characteristics (recipient or donor) nor transplant characteristics have proven to be reliable predictors of SOS (2, 14, 17, 18). Conforming to FDA/NIH-BEST (biomarkers, endpoints, and other tools) recommendations, risk biomarkers are defined as assays that are associated with an increased susceptibility of developing a condition in an individual who does not yet have clinical evidence of that condition (19). Across multiple studies, only a few risk biomarkers for SOS have been identified, but mostly in adults and in retrospective sets, which has several potential limitations that can be addressed in a multicenter prospective study. The 2014 NIH Consensus Development Project further provided a framework for the development of biomarkers into clinical practice, with a critical step of validation in a prospective “real-world” cohort (20). To establish the more granular positive predictive value (PPV) and negative predictive value (NPV), biomarker cutpoints need to be validated in a prospective study (20, 21).

L-ficolin, hyaluronic acid (HA), and stimulation 2 (ST2) were identified and validated in 3 retrospective cohorts as risk biomarkers for SOS (22). An endothelial activation and stress index assessed on the day of transplantation was significantly associated with SOS incidence (23). However, PPV and NPV values were not evaluated in these studies, and therefore the biomarkers were not qualified (19, 23, 24).

Herein, we prospectively assessed L-ficolin, HA, and ST2 at 2 early time points following HCT in a multicenter pediatric cohort. The optimal biomarker cutpoints were determined by exhaustive grid search and Youden’s index using biomarker data from all retrospective cohorts from our previously published study (22). The identified optimal biomarker cutpoints were then applied in our prospective “real-world multicenter” cohort to assess their NPV and PPV as risk biomarkers for SOS for future use in biomarker-based preemptive treatment of SOS. It is important to note that this study is, to the best of our knowledge, the first prospective biomarker analysis reported in the field of HCT.

## Results

### Participant demographics.

**Figure 1:**
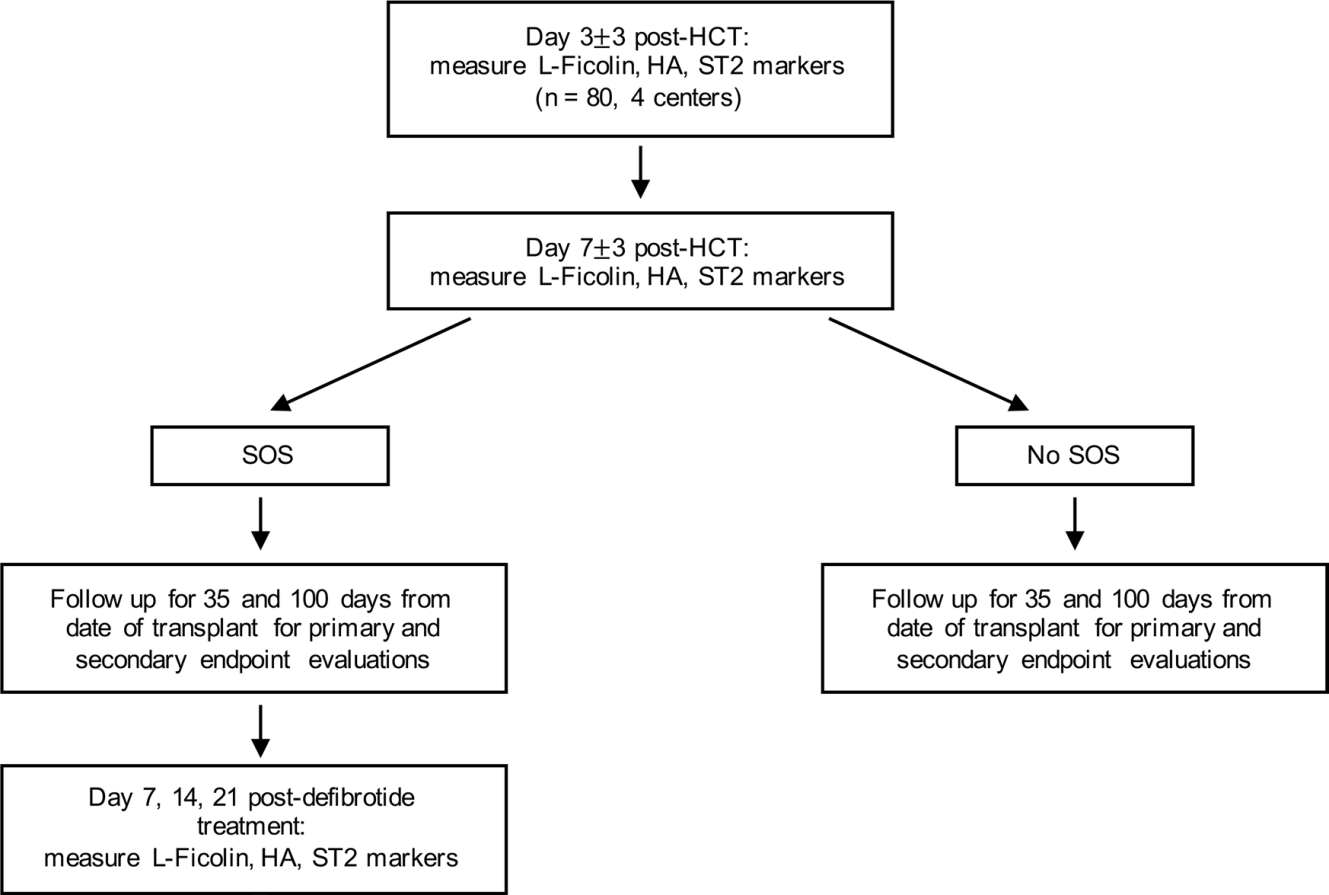
Study design. Workflow illustrating the study population, biomarkers measurements, and time points.

Eighty pediatric patients were prospectively accrued from 4 US academic health centers: Indiana University School of Medicine, Texas Children’s Hospital, University of Michigan, and Children’s National Medical Center. This trial was registered at www.clinicaltrials.gov as NCT03132337 with detailed inclusion and exclusion criteria. The study design is summarized in Figure 1. To allow for better accrual in mid-size pediatric centers, criteria were more permissive than the published pediatric defibrotide prophylaxis study (10). A sample size of 80 was estimated based on the placebo group in the aforementioned European study. Demographics are displayed in Table 1.

A total of 10 of 80 patients (12.5%) developed SOS. The median day after HCT of SOS onset was 19 (range 9–34, Table 2). Using the category of age, children younger than 3 years old were overrepresented in the SOS group (*P* = 0.016, Table 1). There was overrepresentation of cord transplants (*P* = 0.038) among patients with SOS. Graft-versus-host disease (GVHD) prophylaxis with anti–thymocyte globulin (ATG) (*P* = 0.002) was greater among patients with SOS compared with those without. Use of serotherapy with ATG or alemtuzumab was associated with cord transplant (not shown). Although 70% of patients who developed SOS received busulfan in the targeted exposure range (AUC > 900), no significant difference was reached for conditioning regimen. Day 100 OS for the whole cohort was 91% (95% CI 82%–96%, Supplemental Figure 1; supplemental material available online with this article; https://doi.org/10.1172/jci.insight.168221DS1). Causes of death of SOS patients was MOF in all cases. Among non-SOS patients, 1 patient developed idiopathic pneumonia syndrome (IPS), and 1 had relapse of the underlying malignant disease (Table 2 footnote).

### Biomarkers for SOS risk.

Levels of 3 biomarkers (L-ficolin, HA, and ST2) previously identified in a proteomic study (22) were measured in plasma from 80 patients at 2 time points (days 3 and 7 after HCT). Of note, ST2 was selected over VCAM due to unpublished preliminary preclinical and clinical data showing its early relevance in endothelial damage following HCT. VCAM was not selected due to the poor performance of the ELISA with wide intra- and inter-assay variability, which was not seen with L-ficolin, HA, and ST2 ELISAs. Descriptive statistics and correlation among the biomarkers are shown in Supplemental Tables 3 and 4. L-ficolin levels were significantly and inversely correlated with HA (*P* = 0.0025 on day 3 and 0.0001 on day 7) but not ST2, and HA levels were positively correlated with ST2 (*P* < 0.0001 on day 3 and < 0.0001 on day 7), suggesting that the biomarkers represent different pathogenesis pathways. Expectedly, each marker was highly correlated between their values on day 3 and day 7, suggesting that 1 measurement might suffice.

We next calculated the AUCs of the ROCs on day 3 and day 7 after HCT for each marker (Supplemental Figure 2). The 3 biomarkers on day 3 showed AUCs between 0.69 and 0.78 and slightly lower on day 7, between 0.57 and 0.77. We therefore hypothesized that the 3 biomarkers for risk evaluation of developing SOS would be most informative on day 3 after HCT and is also concurrent with the peak of endothelial damage.

**Figure 2:**
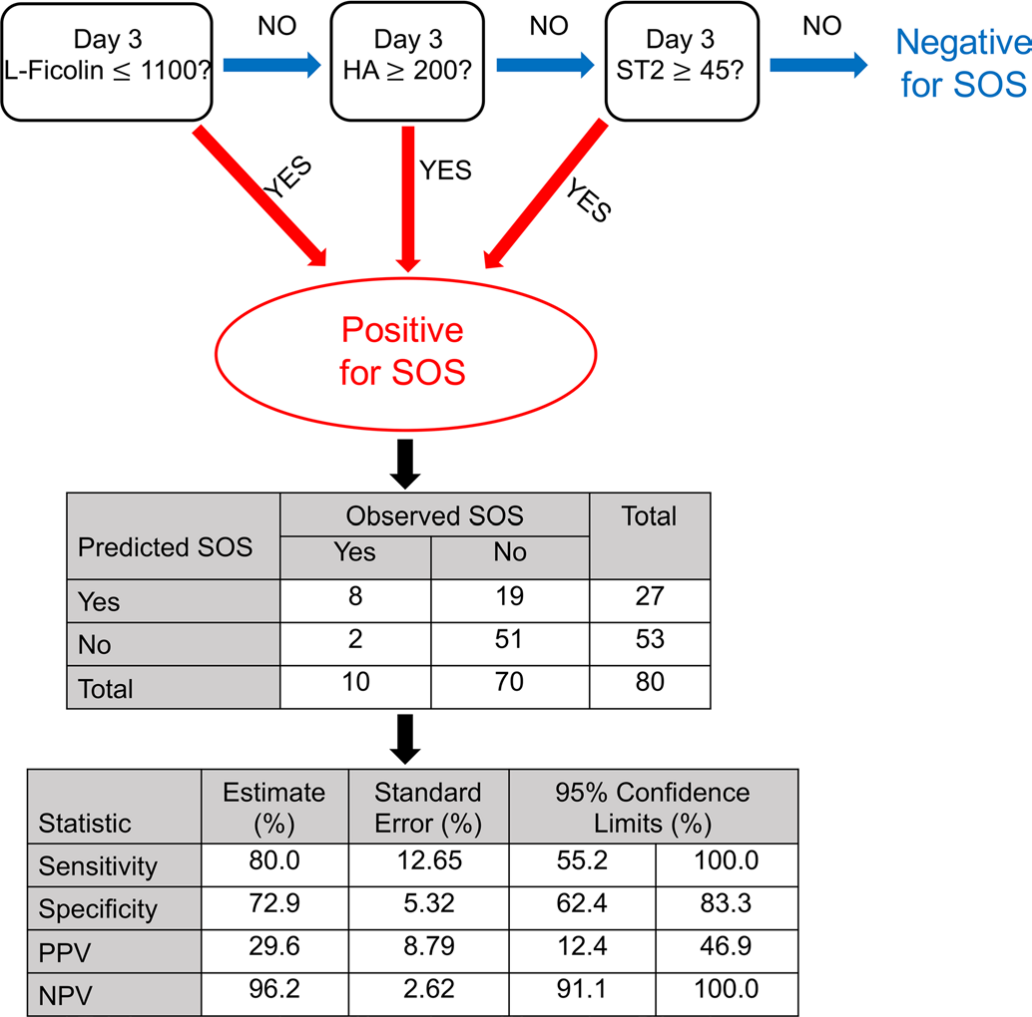
Diagram of the optimal algorithm for risk prediction of SOS, its confusion matrix, sensitivity, specificity, PPV, and NPV.

Optimal cutpoints were determined by exhaustive grid search and Youden’s index based on a previous retrospective study. The retrospective study included biomarker data from 3 cohorts of matched cases (*n* = 34) and controls (*n* = 31). Cutpoints for L-ficolin, HA, and ST2 were 1100, 200, and 45 ng/mL, respectively. Based on these cutpoints, we developed an algorithm for risk prediction of SOS interrogating L-ficolin first, advancing to HA second, and ST2 last. Using this retrospective data, sensitivity, specificity, PPV, and NPV based on the algorithm were 64.7%, 74.2%, 73.3%, and 65.7%, respectively (Supplemental Table 5). When the algorithm was applied to our prospective cohort, sensitivity, specificity, PPV, and NPV for the development of SOS were 80%, 72.9%, 29.6%, and 96.2%, respectively (Figure 2). Regarding the sensitivity, specificity, PPV, and NPV of individual biomarkers using each cutpoint for the development of SOS in the prospective cohort, there was at least 40%, 82.9%, 33.3%, and 91.3% sensitivity, specificity, PPV, and NPV, respectively (Supplemental Table 6). Of note, this algorithm showed the best performance as compared with 2 other statistical analyses using (a) highest sensitivity for at least 50% specificity and (b) regression tree (not shown). Importantly, both false positive rate and false negative rates using the algorithm were low, at 27.1% and 20.0%, respectively.

**Figure 3:**
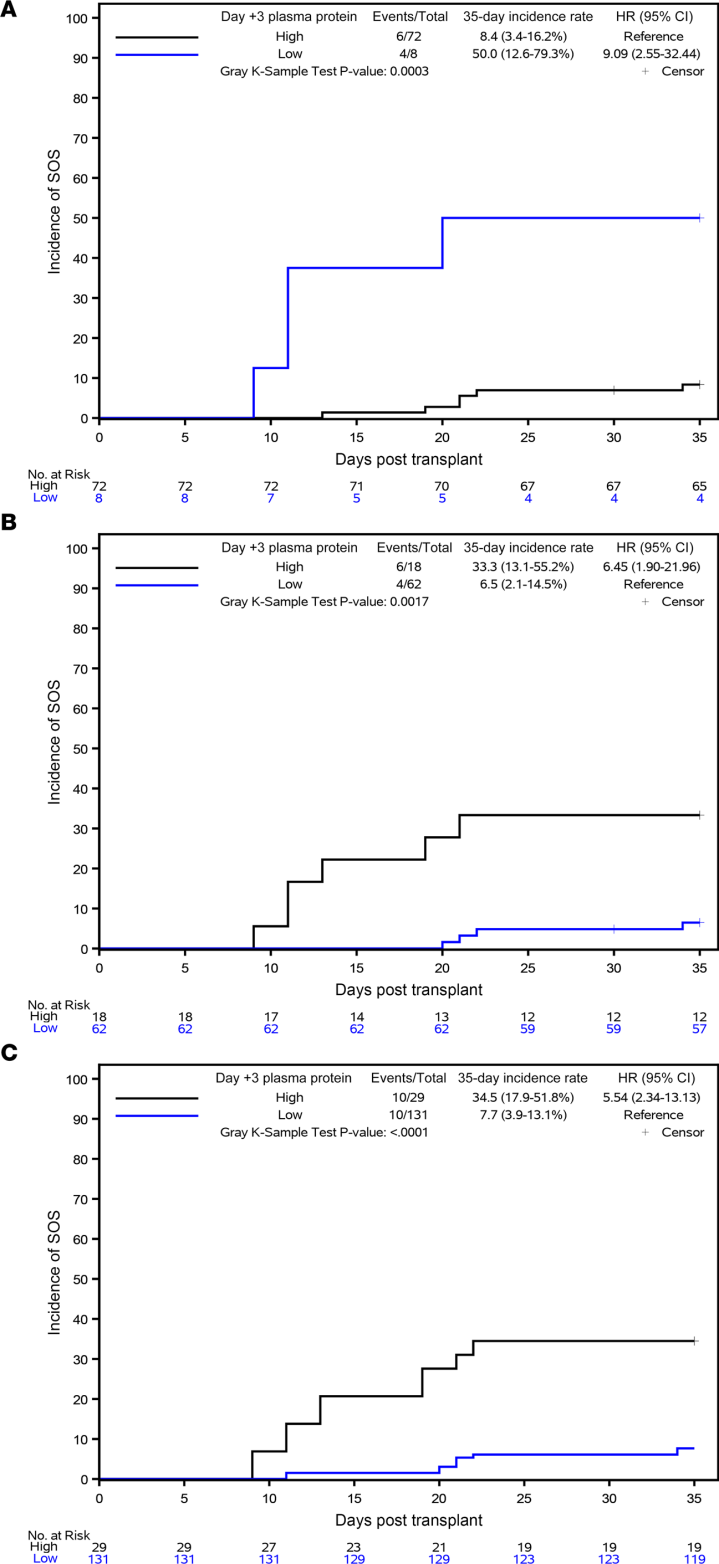
SOS cumulative incidence by day 35 stratified by high and low day 3 risk biomarkers using optimal cutpoints chosen by Youden’s index. (**A**) L-ficolin. (**B**) HA. (**C**) ST2.

**Figure 4:**
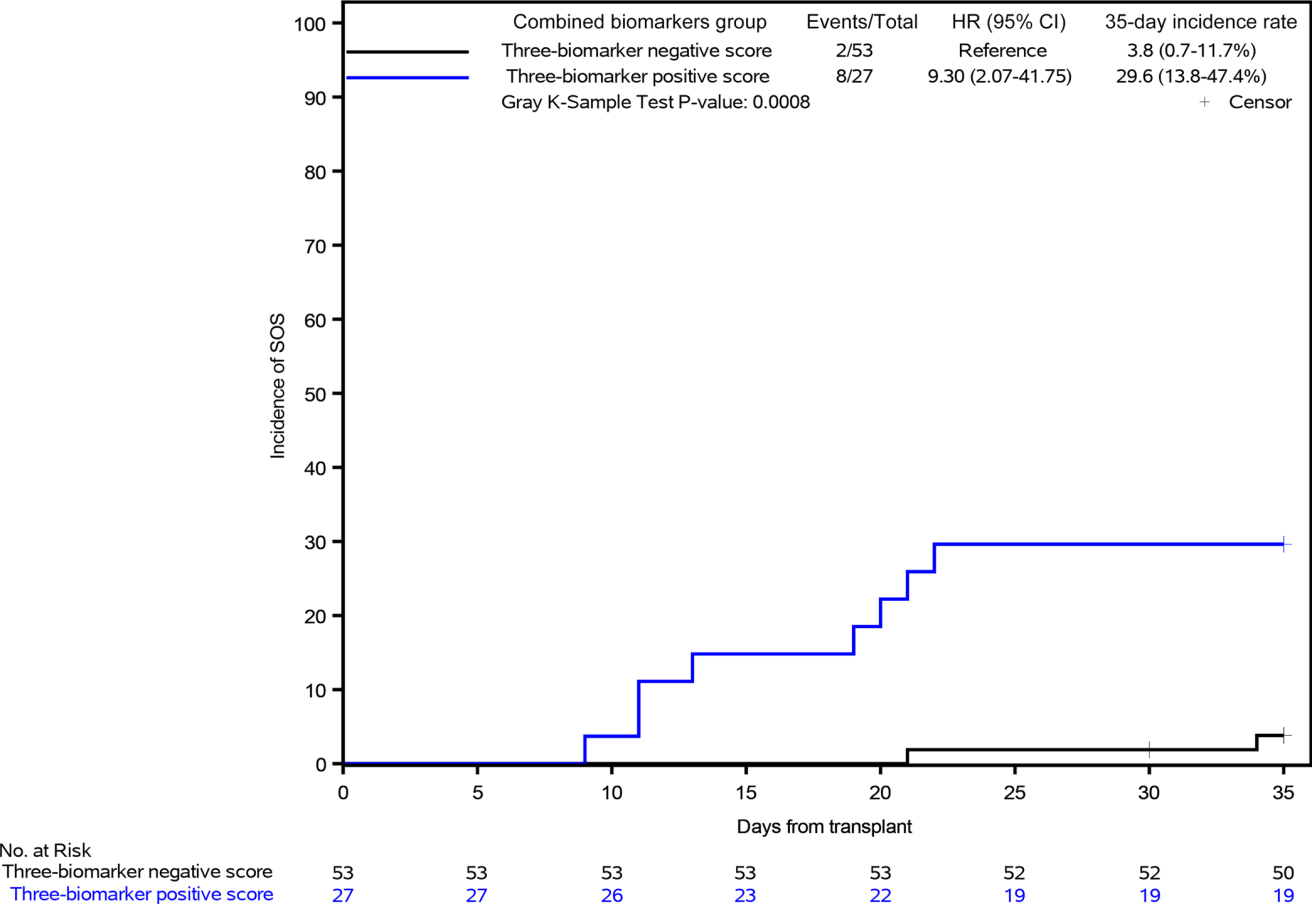
Cumulative incidence of SOS stratified by the 3-biomarker score.

For clinical applicability, biomarkers were then dichotomized into high- and low-risk groups based on cutpoints determined above. Cumulative incidence curves of SOS stratified by day 3 biomarkers were generated individually. Low levels of L-ficolin (<1100 ng/mL) and high levels of HA (>200 ng/mL) and ST2 (>45 ng/mL) were associated with a greater cumulative incidence of SOS. As compared with patients with high L-ficolin values, patients with low L-ficolin values were 9.1 times as likely to develop SOS (95% CI 2.6–32.4, *P* = 0.0003). High HA and high ST2 were also associated with SOS (HA: HR, 6.5; 95% CI 1.9–22.0, *P* = 0.0017; ST2: HR, 5.5; 95% CI 2.3–13.1, *P* < 0.0001) (Figure 3). To investigate the effect of the combination of the 3 biomarkers on SOS risk, the 3 day 3 biomarkers dichotomized as above were incorporated into a Cox proportional hazards regression model to create a 3-biomarker score. Low L-ficolin had a β estimate of 2.47 (95% CI 0.85–4.10). High HA and ST2 had a β estimate of 1.12 (95% CI –0.24 to 2.48) and 2.11 (95% CI 0.56–3.67), respectively (Table 3). Combined biomarkers were divided into 2 groups: 3-biomarker positive score and 3-biomarker negative score, based on the score in Cox’s proportional hazards regression analysis. Compared with patients with 3-biomarker negative score, patients with 3-biomarker positive score were 9.3 times more likely to develop SOS (95% CI 2.1–41.8, *P* = 0.0008) (Figure 4).

### Biomarkers for prognosis of day 100 OS.

**Figure 5:**
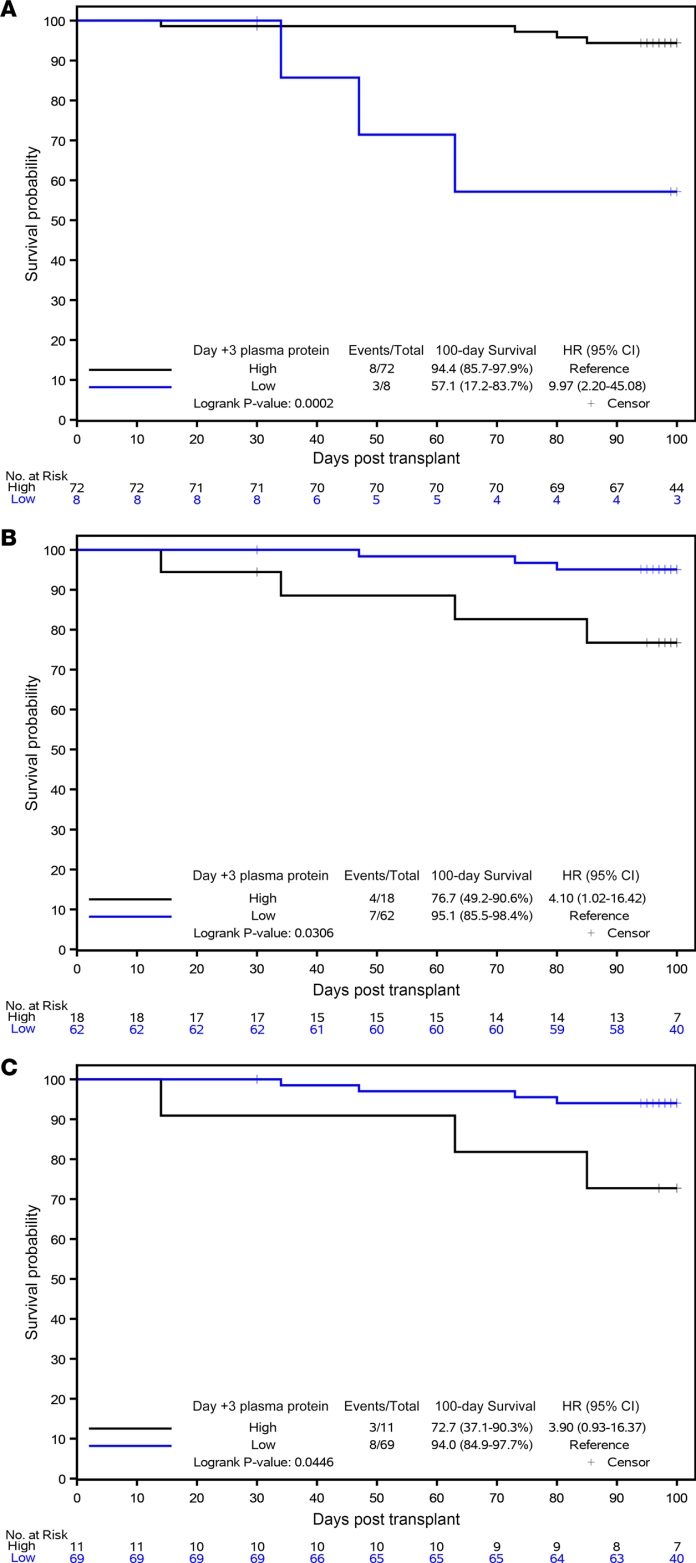
Day 100 OS stratified by high and low day 3 risk biomarkers using optimal cutpoints chosen by Youden’s index. (**A**) L-ficolin. (**B**) HA. (**C**) ST2.

**Figure 6:**
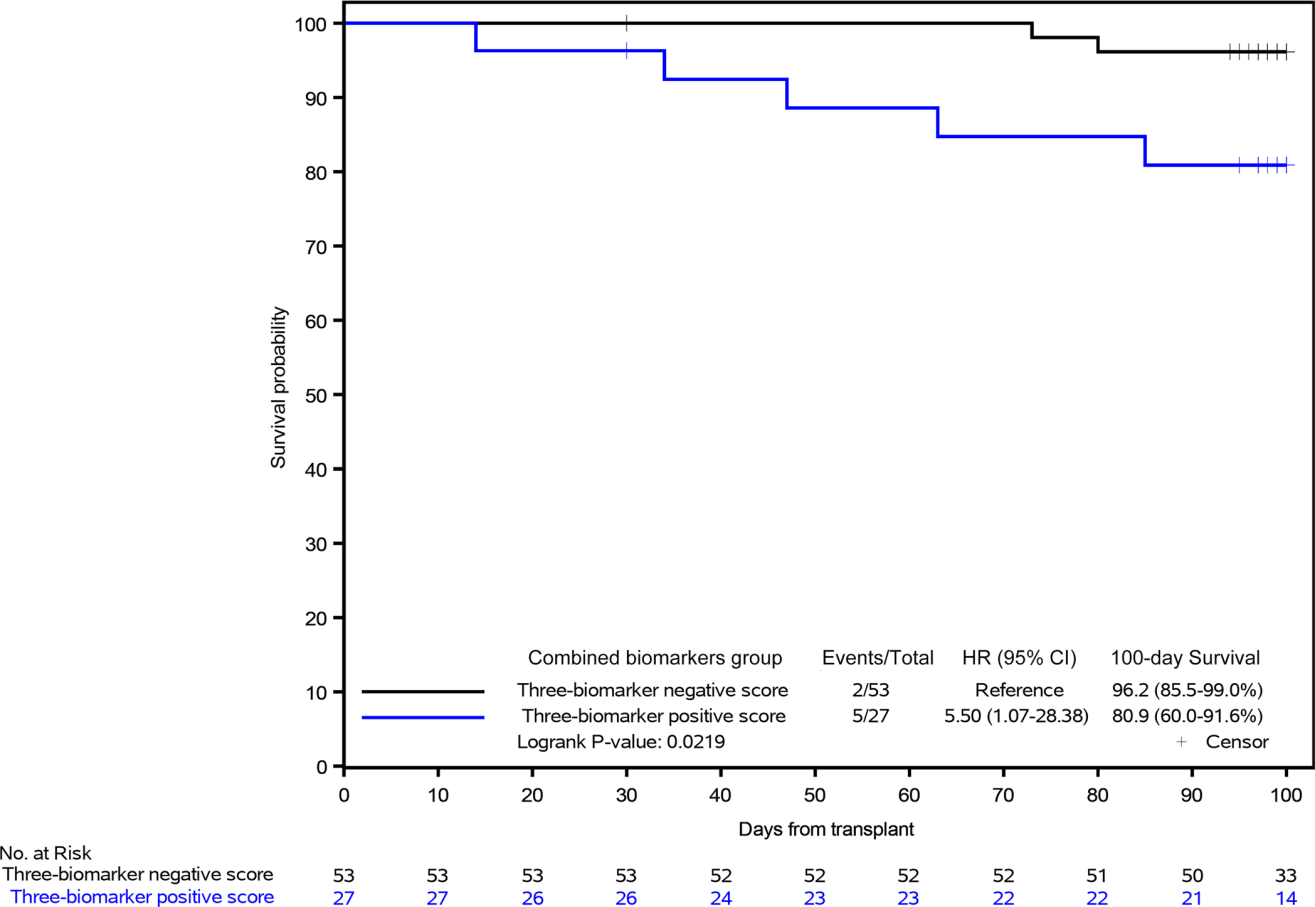
Kaplan-Meier estimates of OS stratified by the 3-biomarker score.

Next, we examined the ability of L-ficolin, HA, and ST2 on day 3 to predict OS by day 100 after HCT using the same cutpoints as for SOS risk. Patients with low L-ficolin, high HA, and high ST2 on day 3 had a lower OS on day 100 — L-ficolin: HR, 10.0 (95% CI 2.2–45.1), *P* = 0.0002; HA: HR, 4.1 (95% CI 1.0–16.4), *P* = 0.031; and ST2: HR, 3.9 (95% CI 0.9–16.4), *P* = 0.045 (Figure 5). For the combination of the 3 biomarkers on day 3, patients with 3-biomarker positive score had a lower OS on day 100, with HR of 5.5 (95% CI 1.1–28.4, *P* = 0.0219) than those with 3-biomarker negative score (Figure 6). Therefore, even as early as day 3 after HCT, L-ficolin, HA, and ST2 predicted worse survival within 100 days after transplantation.

### Associations of biomarkers with potential confounding and multivariable analysis.

Currently, the diagnosis of SOS includes clinical characteristics and measurement of bilirubin (12, 13). Thus, biomarker levels on day 3 were compared with maximum total serum bilirubin, and day 3 total serum bilirubin using Pearson’s coefficients. Even though both HA and ST2 on day 3 were correlated with maximum total serum bilirubin, only ST2 was correlated with total bilirubin measured on day 3 (Supplemental Tables 7 and 8). This suggests that early measurement of ST2 may represent an early liver damage marker. Similarly, when other liver function parameters (alkaline phosphatase [ALP], aspartate aminotransferase [AST], and alanine aminotransferase [ALT]) were measured and correlated to the day 3 biomarkers, only ST2 was slightly associated with ALP, while there was no correlation between any biomarker and AST and ALT on day 3 (Supplemental Tables 9–11), indicating that ST2 might correlate with early bile duct obstruction.

Considering that several other potential complications could occur during the first 35 days, such as cytokine storm (CRS), even if subclinical; thrombotic microangiopathy (TMA), which is typically accompanied by endothelial damage like SOS; IPS; GVHD; and infections, we examined the correlation of the day 3 SOS biomarkers with these 4 potential early complications. Demographics for these other complications are shown in Supplemental Table 12.

As we did not observe any clinical CRS, we used 2 key inflammatory cytokines after HCT (interleukin 6 [IL-6] and tumor necrosis factor receptor 1 [TNFR1]) as surrogates for subclinical CRS. Both day 3 and day 7 TNFR1 levels displayed significant differences between patients with SOS versus without, but IL-6 was not different between groups (Supplemental Table 3). We further correlated elevated TNFR1 with day 3 SOS biomarkers and found it was associated with HA and ST2, but not with L-ficolin (Supplemental Table 13). This may imply that early increased levels of HA and ST2 are indicators of elevated inflammation as well.

Since TMA shares certain features with SOS, including platelet refractoriness and fluid retention (25), we examined the association between day 3 biomarker levels and TMA. Only 2 patients developed TMA, 1 in the SOS group and 1 in the no-SOS group, and no association between biomarker levels and TMA was found (Supplemental Table 14).

SOS can cause rapid weight gain, resulting in respiratory failure, which is also a feature of IPS. Therefore, we examined biomarker levels on day 3 for an association with IPS. Only 2 patients had IPS, 1 in the SOS group and 1 in the no-SOS group, and only L-ficolin was associated with IPS (*P* = 0.032) (Supplemental Table 15).

Given that SOS pathogenesis is a combination of endothelial damage and leukocyte infiltration due to alloreactivity, we investigated biomarker association with overall GVHD grades. Two (20%) patients developed GVHD in the SOS group and 12 (19.3%) developed GVHD in the no-SOS group (Supplemental Table 12). L-ficolin was associated with GVHD, while HA and ST2 were not (Supplemental Table 16). Of note, neither day 3 IL-6 nor day 3 TNFR1 was associated with GVHD (Supplemental Table 17).

Infections commonly occur after HCT; however, there was no difference between patients with SOS versus those without in the rate of infections of grade 3 or higher (Supplemental Table 12), nor was there an association for any biomarkers on day 3 with infection (Supplemental Table 18).

We next calculated associations between high and low biomarkers and SOS incidence in univariate and multivariate Cox proportional hazards regression models (Table 4). All 3 biomarkers were significantly associated with SOS incidence in univariate Cox analysis, and L-ficolin and ST2 remained significant in multivariate analysis after adjustment for the 3 significant clinical covariates between SOS-positive and -negative groups. Subcategorized age (all), transplant source, and GVHD prophylaxis were not significant in the multivariate analysis. However, in multivariate analysis, younger patients (0 to <3 years old) were at higher risk of developing SOS compared with older patients (≥10 to <16 years old) (*P* = 0.037) (Table 4). Since biomarker values were not different in the 4 age categories in the recipients who did not develop SOS (Supplemental Table 19), the correlation seen with age may be due to the higher rate of SOS in younger children rather than an age effect itself. HA had an increased HR that did not reach significance in multivariate analysis, suggesting it is strongly correlated with the 2 other biomarkers, as shown in Supplemental Table 4. In multivariate analyses, patients with low L-ficolin or high ST2 had an independently increased risk of developing SOS, with *P* values of 0.023 and 0.008, respectively (Table 4). These data suggest that L-ficolin and ST2 biomarkers measured as early as day 3 after HCT are independent predictors of future development of SOS in multivariate analysis using this contemporary cohort.

### Monitoring biomarkers following defibrotide treatment.

**Figure 7:**
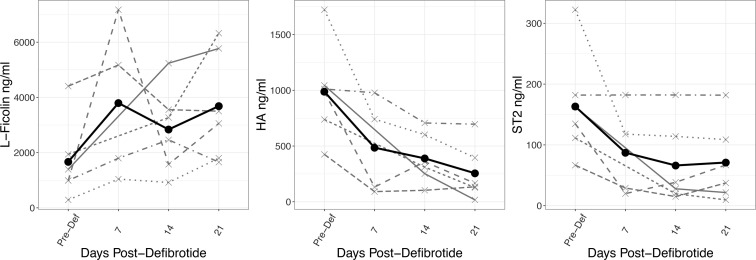
Biomarker changes after defibrotide treatment.

Six out of the 10 patients diagnosed with SOS were treated with defibrotide, and the 3 biomarkers were measured before defibrotide and 7, 14, and 21 days after defibrotide initiation. Figure 7 shows the trend in changes from before defibrotide to day 21 after defibrotide individually and on average. We calculated the rate of change for biomarkers after defibrotide treatment using general linear mixed-effect models and found a significant increase in L-ficolin values (+528 ng/mL, *P* = 0.039) and a decrease in HA and ST2 values (HA, –233 ng/mL, *P* = 0.019; ST2, –30 ng/mL, *P* = 0.037) (Table 5). These data suggest that following treatment with defibrotide, biomarker values normalized toward values seen in patients without SOS.

## Discussion

Early identification with objective proteomic risk biomarkers may improve monitoring and management of SOS, with the goal to decrease risk of MOF. Because we have previously identified and validated L-ficolin, HA, and ST2 as SOS risk biomarkers, we next conducted measurements of L-ficolin, HA, and ST2 on days 3 and 7 after HCT in a prospective cohort of 80 pediatric patients to evaluate NPV and PPV of these risk biomarkers for SOS occurrence, as recommended in the 2014 NIH Consensus Development Project on biomarkers (20, 21). Further, these 3 assays have been verified for analytic validity (precision/accuracy) on 1 platform and have been validated in Clinical Laboratory Improvement Amendments (CLIA) settings. Although VCAM1 was a risk biomarker in the retrospective cohorts, it was not pursued as a qualifying assay due to the poor intra- and interassay variabilities that assess the reproducibility of the ELISA itself.

To our knowledge, this is the only prospective pediatric study conducted to assess SOS risk biomarkers. Identification of optimal cutpoints for the 3 biomarkers was performed solely in 1 retrospective cohort (22). We found that when the 3 markers were measured prospectively on day 3 after HCT, prior to any clinical signs of SOS, they had sensitivity of 80% and specificity of 73% for estimating risk of SOS development, which achieve similar predictability power than the one estimated in the retrospective cohort that was matched 1 to 1 for cases and controls (22). Importantly, Cox’s proportional hazards regression models showed that the markers predicted SOS independently of clinical covariates that were significantly different in patients with SOS versus without (age, transplant source, and GVHD prophylaxis). Notably, younger age (<3 years) remained significant in multivariate analysis as compared with the 10- to 16-years-old category, underpinning the need to closely monitor SOS in this younger population as has been suggested by the PALISI and PTCTC working group (5). The 3 biomarkers on day 3 also predicted SOS independently of commonly measured laboratory tests of liver function. Using the combination of the 3 biomarkers as a score, recipients with a positive score were 9.3 times more likely to develop SOS (95% CI 2.1–41.8, *P* = 0.0008) (Figure 4). This suggests that L-ficolin, HA, and ST2 individually and dichotomized by high and low thresholds or as a combined 3-biomarker score predict subclinical SOS disease several days before the diagnosis of SOS is made.

These 3 biomarkers do not predict other early complications. Notably, ROC analyses for the 3 biomarkers showed higher AUCs on day 3 compared with day 7, particularly for ST2, which might be explained by the fact that ST2 is a confounding marker of GVHD risk when measured on or after day 7 (26). However, ST2 analyzed as a continuous variable on day 7 was not correlated with GVHD, which is concordant with previous studies that showed that ST2 levels before HCT or earlier after HCT were correlated with nonrelapse mortality (NRM), but association with GVHD per se was either not checked or significant only when landmark analysis included ST2 measurements that were performed on days 7, 14, and 21 (26–28). We further examined in this prospective cohort IL-6 and TNFR1 early after HCT, and although TNFR1 was associated with SOS development and correlated with HA and ST2, neither IL-6 nor TNFR1 was associated with GVHD, as has been shown in a previous prospective pediatric cohort focused on analyzing NRM and GVHD (28). HA and ST2 on day 3 were not significantly associated with TMA, IPS, or infection in this prospective cohort.

Pathogenesis of the 3 markers, although multifactorial, includes endothelial injury. Indeed, L-ficolin, a major plasma complement-activating pattern-recognition lectin, is synthesized in the liver, secreted into the bloodstream, and is involved in homeostatic clearance of mitochondria (29). In SOS patients, its concentrations are decreased, suggesting a homeostatic clearance deficiency. Interestingly, L-ficolin was correlated with GVHD and IPS, suggesting a role in alloreactivity not previously described. L-ficolin has been shown to bind to Toll-like receptor 4 on macrophages and dendritic cells and promote their antigen presentation to CD8^+^ T cells (30). HA is produced by mesenchymal cells, and its levels are maintained by a receptor-dependent removal mechanism in the sinusoidal endothelial cells of the liver. Systemic HA levels are regarded as a direct marker of hepatic sinusoidal endothelial cell function and elevated concentrations are associated with SOS (22, 31). ELISA measures soluble ST2, acting as a decoy receptor for IL-33 (32). In an HCT preclinical model, ST2 has been shown to be initially secreted by endothelial cells and later by alloreactive T cells (33). These biomarkers representing novel biological pathways in SOS could also be therapeutic targets.

We also interrogated the markers as predictors of OS within 100 days after HCT. L-ficolin, HA, and ST2 were also found to be prognostic markers of OS using cutpoints as risk biomarkers. Although the SOS incidence was low, the severity and development to MOF in SOS patients was high. Using the 3-biomarker score, recipients with a positive score were 5.5 times more likely to die by day 100 (95% CI 1.1–28.4, *P* = 0.0219) than those with a 3-biomarker negative score (Figure 6).

Six out of the 10 patients diagnosed with SOS received defibrotide, while the other patients were not able to receive the treatment due to contraindications, particularly bleeding. This highlights the importance of early diagnosis based on revised criteria and early intervention (15, 16, 34). Five of the SOS patients died within 100 days. In 2 randomized trials of defibrotide for the prevention of SOS, OS was not different between placebo and defibrotide groups (10, 11). The 3 markers were further tested as potential monitoring markers of response to defibrotide treatment and showed a trend toward normalization to non-SOS patient levels as early as 7 days after treatment. If validated in a larger cohort, it could potentially justify defibrotide treatment for a shorter time course than the current 21 days.

This study has its limitations, however. In this contemporary prospective cohort of 80 pediatric patients with known pre-HCT risk factors for SOS, 10 patients developed SOS, resulting in a 12.5% incidence that is lower than the 20% reported in the placebo group from the defibrotide prophylaxis study, which might be explained by more the permissive inclusion criteria as compared with the defibrotide study (10). It may also be explained by the changes to conditioning regimens and better supportive care since 2012. Because PPV is dependent on the disease incidence, this might explain its lower value. A recent analysis of data from 15 pediatric centers associated the magnitude of intravenous busulfan exposure with the development of SOS in children and young adults undergoing myeloablative allogeneic HCT (35). A Center for International Blood and Marrow Transplant Research study found an association between SOS risk and centers performing busulfan-based myeloablative conditioning regimens guided by pharmacokinetic monitoring rather than using the target level (36). Although preparative regimen was not a significant parameter in our study, we did not report AUC in this deidentified cohort and adjustment for this parameter was not possible. In our study, SOS diagnosis was determined using the modified Seattle criteria, as they were the criteria used for defibrotide FDA approval in 2017. However, anicteric SOS forms that are frequent in children may have been underestimated (15, 16). Newer diagnostic scorings were not looked at retrospectively in this deidentified prospective cohort. Although there was no association between biomarker levels and TMA in this cohort, it is worth noting that TMA is a challenging clinical diagnosis and not all centers routinely screen for it. Further, in a recent study administering defibrotide for prophylaxis of TMA, high ST2 correlated with SOS diagnosis and with the only patient who died (37). Although we had 27% false positives for a day 3 test, on average 15 days before the diagnosis, the possibility of overtreatment here is not a significant concern for 3 reasons: (a) we have used stringent criteria for the diagnosis of SOS, and it is likely that some of these false positives are anicteric SOS, which is frequent in children, and we will be able to use the new criteria in an interventional preemptive trial; (b) defibrotide treatment has low to no toxicity, particularly when given earlier; and (c) the cost of the drug is a less of a concern in a pediatric population. Finally, while the biomarker cutpoints identified in this study are promising and established in a prospective cohort, their validity will be only demonstrated if their implementation in an interventional preemptive trial (Supplemental Figure 3) will decrease SOS incidence as compared with the 12.5% incidence found herein.

We conclude that in this prospective cohort study, 3 plasma biomarkers of endothelial damage measured noninvasively, 3 days after HCT, were associated with SOS occurrence and OS. Assessing these markers could stratify patients at high risk for SOS who may benefit from preemptive intervention with defibrotide.

## Methods

### Patients.

Pediatric patients (up to 22 years old) of any sex, race, and ethnicity undergoing HCT for any indication who fulfill clinical criteria for high-risk of SOS at enrollment (i.e., history of hepatic disease, conditioning with busulfan or total body irradiation, ≥2 HCTs) were eligible for enrollment. Patients were recruited across 4 centers from 2017 to 2021. A complete list of inclusion and exclusion criteria is provided in the Supplemental Methods. SOS was diagnosed based on modified Seattle criteria (12). Supplemental Table 1 shows the SOS severity scale used in the trial (38).

### Sample collection, processing, and ELISA.

Plasma samples were prospectively collected on days 3 and 7 after HCT, prior to the onset of SOS. A window of ±3 days was authorized to avoid shipment on weekends; however, day 0 samples were taken several hours after the graft transfusion. ELISA procedures and parameters are described in Supplemental Methods and Supplemental Table 2.

### Data and materials availability.

Biomarker raw data are available through a material transfer agreement with the Medical University of South Carolina; direct all inquiries to SP. All detection tools are available through commercial vendors. All data associated with this paper are present in the paper and/or in the supplemental materials.

### Statistics.

Differences in patient group characteristics were assessed using either Fisher’s exact test or Wilcoxon’s rank-sum test. Differences for biomarkers and liver functions between SOS yes or no were checked by 2-sample *t* test or Wilcoxon’s rank-sum test. Correlations between biomarkers and liver functions or inflammatory cytokines were measured using Pearson’s correlation coefficients. ROC curves were generated on days 3 and 7. The optimal biomarker cutpoints were obtained from biomarker values of all 3 cohorts from our previous retrospective study (22) based on an exhaustive grid search and Youden’s index selection. First, each biomarker was set to be in a clinically relevant range of values (L-ficolin: 500 to 1200 ng/mL; HA: 50 to 220 ng/mL; ST2: 30 to 50 ng/mL). We then calculated Youden’s index for each combination of L-ficolin, HA, and ST2. The cutpoints were determined by searching the combination that could reach the higher Youden’s index. Kaplan-Meier–based cumulative incidence curves for SOS by day 35 and OS curves by day 100 were produced with day 3 individual biomarker or combined 3 biomarkers using the aforementioned cutpoints to determine high- and low-risk groups. Cox’s proportional hazards regression score was used to generate 2 combined biomarker groups for the cumulative incidence and OS curves. First, 3 binary day 3 biomarkers (L-ficolin low/high, HA high/low, and ST2 high/low were coded as 1/0) were incorporated into Cox’s proportional hazards regression to obtain the β estimates for each biomarker. Second, score was defined as β × *x* for each patient, where *x* is the covariate. Third, 2 combined biomarker groups, 3-biomarker positive score and 3-biomarker negative score, were formed according to score greater than 0 and score equal to 0, respectively. Comparison between groups was evaluated using Gray’s K-sample tests or log-rank tests. Associations between biomarkers and inflammatory cytokines, TMA, IPS, GVHD, and infections were examined by logistic regression models. Univariate and multivariate Cox regression analyses evaluated the effect of day 3 biomarkers adjusted for significant clinical characteristics. For biomarker monitoring after defibrotide initiation, we estimated the rate of change by fitting general linear mixed random intercepts and slopes effect models (using PROC MIXED) with an unstructured correlation for time. Statistical significance was defined as a *P* value of less than 0.05.

### Study design and approval.

The study design and samples collection are summarized in Figure 1. The study was approved by the Institutional Review boards of all institutional participating centers. Written informed consent was obtained from all patients or their legal guardians.

## Author contributions

SP designed and oversaw the study. YH, HL, and SP analyzed data and ensured data integrity. EDPA, RAK, JR, JLS, and SWC oversaw patient sample collection and processing at the respective centers. AB, BPD, AM, KBV, and SP were responsible for centralized sample processing, storage, and proteomic analysis with ELISAs. All authors reviewed and provided feedback on the initial study design, collected data, interpreted results, and read, edited, and approved the final version of the manuscript.

## Supplementary Material

Supplemental data

## Figures and Tables

**Table 1:**
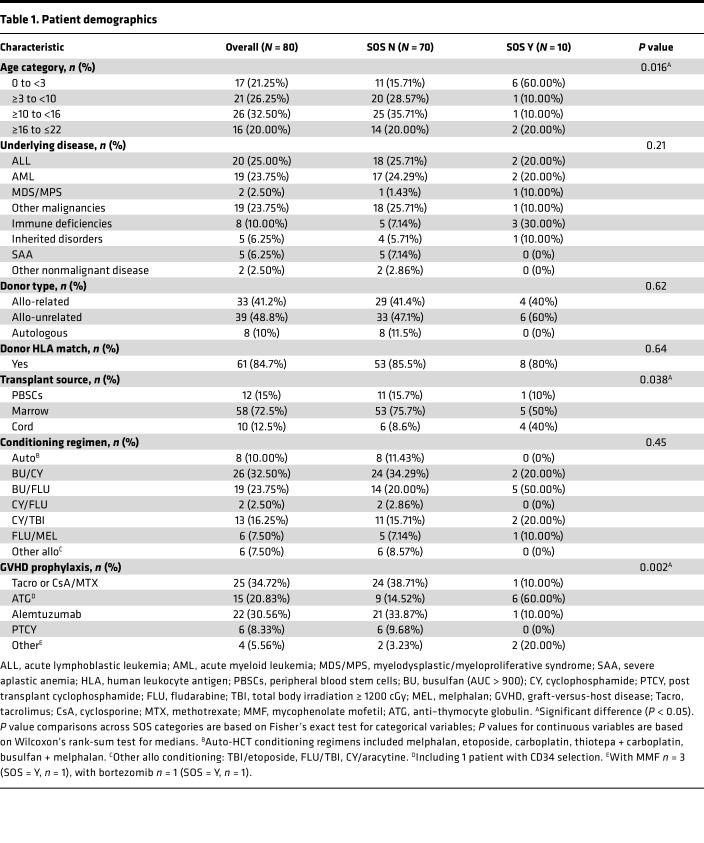
Patient demographics

**Table 2:**
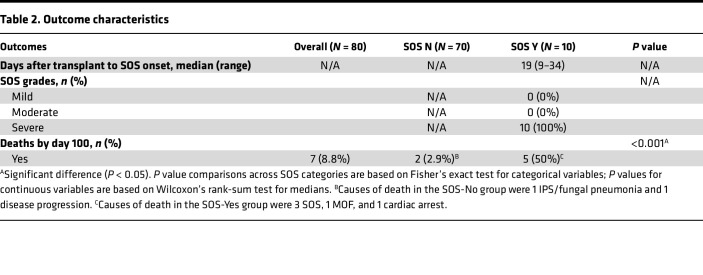
Outcome characteristics

**Table 3:**
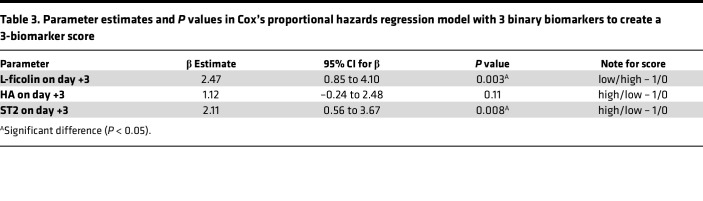
Parameter estimates and *P* values in Cox’s proportional hazards regression model with 3 binary biomarkers to create a 3-biomarker score

**Table 5:**
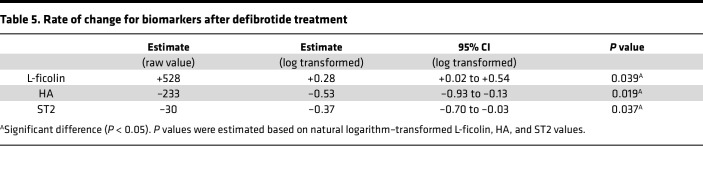
Rate of change for biomarkers after defibrotide treatment

**Table 4:**
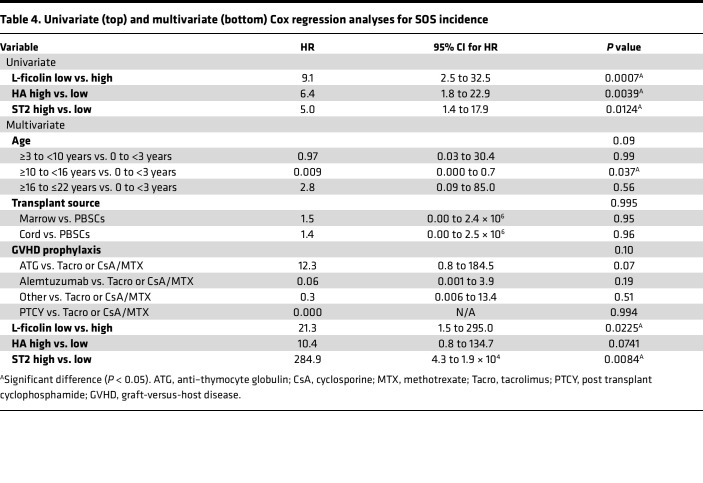
Univariate (top) and multivariate (bottom) Cox regression analyses for SOS incidence

## Version 1 04/18/2023

In-Press Preview

## Version 2 05/22/2023

Electronic publication
