# Supplementary material for: Prospective assessment of risk biomarkers of sinusoidal obstruction syndrome after hematopoietic cell transplantation
Source: JCI Insight. 2023 May 22;8(10):e168221. doi: 10.1172/jci.insight.168221 (PMC10322680; doi:10.1172/jci.insight.168221)

Supplemental materials to:

Prospective Assessment of Risk Biomarkers of Sinusoidal Obstruction Syndrome After Hematopoietic Cell Transplantation

Table of Contents

Table S1. SOS Severity Scale

Table S2. Details of ELISAs used for protein testing

Table S3. Descriptive Statistics

Table S4. Pearson Correlation between Biomarkers

Table S5. Sensitivity, specificity, PPV, and NPV using optimal cutpoints in retrospective cohort

Table S6. Sensitivity, specificity, PPV, and NPV using optimal cutpoints in prospective cohort

Table S7. Correlation between biomarker levels and maximum total serum bilirubin

Table S8. Correlation between biomarker levels and total bilirubin at day 3

Table S9. Correlation between biomarker levels and alkaline phosphatase at day 3

Table S10. Correlation between biomarker levels and AST at day 3

Table S11. Correlation between biomarker levels and ALT at day 3

Table S12. Demographics for other post-HCT complications

Table S13. Correlation between biomarker levels and TNFR1 at day 3

Table S14. Association between biomarker levels and thrombotic microangiopathy (TMA)

Table S15. Association between biomarker levels and idiopathic pneumonia syndrome (IPS)

Table S16. Association between biomarker levels and overall graft-versus-host disease (GVHD) severity

Table S17. Association between IL-6 and TNFR1 levels and overall graft-versus-host disease (GVHD) severity

Table S18. Association between biomarker levels and infections

Table S19. Descriptive statistics of biomarkers in subcategorized age in patients without SOS.

Figure S1. Kaplan-Meier estimates of overall survival (OS) on day 100 post-transplant

Figure S2A. ROC curves for day+3 biomarkers

Figure S2B. ROC curves for day+7 biomarkers

Figure S3. Proposed preemptive trial design

Supplementary Methods

Study Patients.

Detailed eligibility criteria for the study are described below

Inclusion Criteria

Age ≤ 25 years undergoing HCT for any reason who fulfill any ONE of the following criteria:

1. History of hepatic disease as defined by:
   a. Viral hepatitis (i.e., hepatitis C virus [HCV])
   b. Liver tumor before HCT
   c. Hepatic fibrosis or cirrhosis before HCT as proven by liver biopsy
   d. High aspartate aminotransferase (AST) (> 2x ULN) before HCT (pre-transplant evaluation)
   e. High alanine transaminase (ALT) (> 2x ULN) before HCT
   f. High bilirubin (> 1.2x ULN) before HCT
2. HCT high-risk features including:
   a. Conditioning with high-risk modalities including:
      i. Busulfan (BU)-containing regimen particularly with oral BU + cyclophosphamide
      ii. TBI-containing regimen, particularly cyclophosphamide + total-body irradiation (TBI)
   b. ≥ 2 HCT
   c. Allo-HCT for leukemia > or = second relapse
   d. Unrelated donor (URD) HCT
   e. Human leukocyte antigen (HLA) mismatch HCT (less than 10 of 10 for bone marrow/peripheral blood stem cell [BM/PBSC] or anything less than 6 of 6 for UCB)
   f. Use of sirolimus + tacrolimus prophylaxis for GVHD
3. High-risk disease states including:
   a. Juvenile myelo-monocytic chronic leukemia (JMML)
   b. Primary hemophagocytic lymphohistiocytosis (HLH)
   c. Adrenoleukodystrophy
   d. Osteopetrosis
4. Other high-risk features including:
   a. Prior treatment with gemtuzumab ozogamicin
   b. Use of hepatotoxic drugs 1 month before HCT and during HCT
   c. Iron overload (i.e., thalassemia/sickle cell) with serum ferritin > 1000ng/ml
   d. Deficit of ATIII, T-PA (i.e., < 30% normal values), and resistance to activated protein C if clinical indication (these values do not have to be specifically checked if no clinical history)
   e. Young age < 2 years but more than 1 month

Exclusion Criteria

Patients who are transplanted but do not fulfill any of the above-mentioned criteria.

ELISA.

Antibody pairs were purchased as described in Supplemental Table 2. Proteins were measured in samples using commercially available enzyme-linked immunosorbent assays (ELISA) and following the manufacturer' recommendations and using a sequential ELISA approach previous described. All samples and standards were tested in duplicate. All washes were performed using the Aquamax 2000 plate washer (Molecular Devices, Sunnyvale, CA). Absorbance was measured immediately after termination of the substrate reaction using a SpectraMax ABS Plus plate reader and results were calculated using SoftMax Pro Version 7.1 (Molecular Devices, Sunnyvale, CA).

Table S1: SOS Severity Scale

Criteria	SOS Grade		
	**Mild**	**Moderate**	**Severe**
**Bilirubin mg/dL**	2.0-3.0	3.1-5.0	> 5.0
**Liver function**	< 3X normal	3-5 normal	> 5X normal
**Weight above baseline**	2%	2.1-5%	> 5%
**Renal function**	normal	< 2X normal	≥ 2X normal
**Rate of change, days**	Slow (> 6)	Moderate (4-5)	Rapid (< 3)

Table S2: Details of ELISAs used for protein testing

Protein name	Description	Commercial ELISA provider	Plasma dilution	LLOD	ULOD
L-Ficolin	Ficolin-2	Hycult Biotech	1:20	16 ng/ml	20000 ng/ml
HA	Hyaluronic Acid	Corgenix	1:1	30 ng/ml	4000 ng/ml
ST2	Stimulation-2, IL-33 receptor	R&D Quantikine	1:50	1 ng/ml	200 ng/ml
IL-6	Interleukin-6	R&D Duoset	1:1	0.5 pg/ml	2000 pg/ml
TNFR1	Tumor Necrosis Factor Receptor -1	R&D Duoset	1:25	100 pg/ml	20000 pg/ml

Table S3. Biomarkers Descriptive Statistics

Variable	SOS Yes (n =10)			SOS No (n =70)			P-value
	N	Mean ± Standard Deviation	Median (Min – Max)	N	Mean ± Standard Deviation	Median (Min – Max)	
**Biomarkers**							
**L-Ficolin Day +3**	10	2399 ± 2036	2002 (158 – 5932)	70	3722 ± 2162	3321 (848– 14373)	0.07*
**HA Day +3**		465 ± 572	216 (67 – 1870)		167 ± 236	78 (17 – 1422)	0.004#†
**ST2 Day +3**		63 ± 65	36 (6 – 202)		26 ± 18	20 (3 – 99)	0.036#†
**L-Ficolin Day +7**	10	2592 ± 2553	1721 (289 – 7176)	69	3596 ± 2048	3197 (357 – 12038)	0.09#
**HA Day +7**		751 ± 745	351 (82 – 2043)		195 ± 218	117 (27 – 1176)	0.005#†
**ST2 Day +7**		72 ± 101	26 (8 – 322)		30 ± 24	21 (6 – 113)	0.47#
**IL6 Day +3**	10	215 ± 509	8 (0 – 1602)	70	37 ± 64	18 (0 – 345)	0.72#
**TNFR1 Day +3**		3913 ± 5609	1809 (824 – 19575)		1044 ± 659	906 (184 – 5242)	<.001#†
**IL6 Day +7**	10	244 ± 657	16 (0 – 2107)	69	53 ± 78	26 (0 – 421)	0.82#
**TNFR1 Day +7**		6948 ± 13725	2330 (1129 – 45816)		1218 ± 576	1086 (184 – 3116)	0.0002#†
**Liver Functions**							
**Bilirubin Day +3**	10	0.63 ± 0.39	0.6 (0.2 – 1.3)	70	0.47 ± 0.35	0.4 (0.0 – 1.5)	0.22#
**AST Day +3**		31 ± 14	31 (12 – 52)		37 ± 27	28 (12 – 154)	0.80#
**ALT Day +3**		33 ± 28	22 (10 – 102)		40 ± 36	26 (5 – 176)	0.71#
**Bilirubin Day +7**	10	0.80 ± 0.66	0.55 (0.2 – 2)	69	0.45 ± 0.26	0.4 (0.0 – 1.4)	0.17#
**AST Day +7**		21 ± 7	22 (12 – 34)		35 ± 34	29 (9 – 253)	0.08#
**ALT Day +7**		21 ± 9	18 (8 – 37)		45 ± 55	23 (5 – 276)	0.24#

Note *: Using the two-sample t-test

Note #: Using the Wilcoxon Rank Sum test

Note †: P-value < 0.05

Table S4. Pearson Correlation between Biomarkers

Pearson Correlation Coefficients<br>Prob > |r| under H0: Rho=0<br>Number of Observations						
	**L-Ficolin Day +3**	**HA Day +3**	**ST2 Day +3**	**L-Ficolin Day +7**	**HA Day +7**	**ST2 Day +7**
**L-Ficolin Day +3**	1.00000<br><br>80	-0.33312<br>0.0025*<br>80	-0.15587<br>0.1674<br>80	0.72525<br><.0001*<br>79	-0.36196<br>0.0010*<br>79	-0.11837<br>0.2988<br>79
**HA Day +3**		1.00000<br><br>80	0.69013<br><.0001*<br>80	-0.33571<br>0.0025*<br>79	0.64033<br><.0001*<br>79	0.70611<br><.0001*<br>79
**ST2 Day +3**			1.00000<br><br>80	-0.19167<br>0.0906<br>79	0.48597<br><.0001*<br>79	0.74086<br><.0001*<br>79
**L-Ficolin Day +7**				1.00000<br><br>79	-0.42077<br>0.0001*<br>79	-0.19647<br>0.0827<br>79
**HA Day +7**					1.00000<br><br>79	0.55123<br><.0001*<br>79
**ST2 Day +7**						1.00000<br><br>79

Note*: p-value < 0.05.

Table S5. Sensitivity, specificity, PPV, and NPV using optimal cutpoints in retrospective cohort

Statistic	Estimate (%)	Standard Error (%)	95% Confidence Limits (%)	
Sensitivity	64.71	8.20	48.64	80.77
Specificity	74.19	7.86	58.79	89.60
Positive Predictive Value	73.33	8.07	57.51	89.16
Negative Predictive Value	65.71	8.02	49.99	81.44

Table S6. Sensitivity, specificity, PPV, and NPV of individual biomarkers using optimal cutpoints in prospective cohort

Biomarker	Statistic	Estimate (%)	Standard Error (%)	95% Confidence Limits (%)	
L-Ficolin	Sensitivity	40.00	15.49	9.64	70.36
	Specificity	94.29	2.77	88.85	99.72
	Positive Predictive Value	50.00	17.68	15.35	84.65
	Negative Predictive Value	91.67	3.26	85.28	98.05
HA	Sensitivity	60.00	15.49	29.64	90.36
	Specificity	82.86	4.50	74.03	91.69
	Positive Predictive Value	33.33	11.11	11.56	55.11
	Negative Predictive Value	93.55	3.12	87.43	99.66
ST2	Sensitivity	40.00	15.49	9.64	70.36
	Specificity	90.00	3.59	82.97	97.03
	Positive Predictive Value	36.36	14.50	7.94	64.79
	Negative Predictive Value	91.30	3.39	84.66	97.95

Table S7. Correlation between biomarker levels and maximum total serum bilirubin

Biomarker and Time Point	n	Pearson's r	p-value
L-Ficolin at Day +3	80	-0.1250	0.27
HA at Day +3	80	0.2468	0.027*
ST2 at Day +3	80	0.4298	<.001*

Note*: p-value < 0.05.

Table S8. Correlation between biomarker levels and total bilirubin at day 3

Biomarker and Time Point	n	Pearson's r	p-value
L-Ficolin at Day +3	80	0.0398	0.73
HA at Day +3	80	0.0430	0.71
ST2 at Day +3	80	0.3066	0.006*

Note*: p-value < 0.05.

Table S9. Correlation between biomarker levels and alkaline phosphatase at day 3.

Biomarker and Time Point	n	Pearson's r	p-value
L-Ficolin at Day +3	79	0.0040	0.97
HA at Day +3	79	-0.0079	0.95
ST2 at Day +3	79	-0.2260	0.045*

Note*: p-value < 0.05.

Table S10. Correlation between biomarker levels and AST at day 3.

Biomarker and Time Point	n	Pearson's r	p-value
L-Ficolin at Day +3	80	-0.1366	0.23
HA at Day +3	80	0.2010	0.07
ST2 at Day +3	80	-0.0433	0.70

Table S11. Correlation between biomarker levels and ALT at day 3.

Biomarker and Time Point	n	Pearson's r	p-value
L-Ficolin at Day +3	80	-0.0790	0.49
HA at Day +3	80	0.1452	0.20
ST2 at Day +3	80	-0.0496	0.66

Table S12. Demographics for other post-HCT complications

Characteristic	Overall N=80	SOS N N=70	SOS Y N=10	P-value
**# of TMA cases n (%)**				0.24
No	78 (97.5%)	69 (98.6%)	9 (90%)	
Yes	2 (2.5%)	1 (1.4%)	1 (10%)	
**# of IPS cases n (%)**				0.26
No	70 (97.2%)	61 (98.4%)	9 (90%)	
Yes	2 (2.8%)	1 (1.6%)	1 (10%)	
**Acute GVHD n (%)**				1.00
No	58 (80.6%)	50 (80.7%)	8 (80%)	
Yes	14 (19.4%)	12 (19.3%)	2 (20%)	
**Days after transplant to GVHD onset Median (range)**	48 (18, 305)	40 (18, 305)	64 (63, 66)	0.08
**GVHD grades n (%)**				0.34
0	58 (80.6%)	50 (80.7%)	8 (80%)	
I- Stage 1-2 Rash and no liver or gut involvement	5 (6.9%)	5 (8.1%)	0 (0%)	
II- Stage 3 rash, or stage 1 liver involvement or stage 1 gut involvement	5 (6.9%)	3 (4.8%)	2 (20%)	
III- None to stage 3 skin rash with stage 2-3 liver, or stage 2-4 gut involvement	4 (5.6%)	4 (6.5%)	0 (0%)	
**Infection grade >= 3 n (%)**				0.12
Bacterial	10 (55.56%)	8 (53.33%)	2 (66.67%)	
Bacterial and Viral	1 (5.56%)	0 (0%)0 (0%)	1 (33.33%)	

Characteristic	Overall<br><br>N=80	SOS N<br><br>N=70	SOS Y<br><br>N=10	P-value
Viral	7 (38.89%)	7 (46.67%)	0 (0%)	

TMA, Thrombotic Microangiopathy; IPS, Idiopathic Pneumonia Syndrome; GVHD, Graft-Versus-Host Disease
Note┼: P-value < 0.05, P-value comparisons across SOS categories are based on fisher's exact test for categorical variables; p-values for continuous variables are based on the Wilcoxon Rank Sum test for median.

Table S13. Correlation between biomarker levels and TNFR1 at day 3

Biomarker and Time Point	n	Pearson's r	p-value
L-Ficolin at Day +3	80	-0.1672	0.14
HA at Day +3	80	0.5029	<.001*
ST2 at Day +3	80	0.7822	<.001*

Note*: p-value < 0.05.

Table S14. Association between biomarker levels and thrombotic microangiopathy (TMA)

Biomarker and Time Point	n	Beta estimate	95% CI		p-value
L-Ficolin at Day +3	80	-0.00002	-0.0007	0.0006	0.95
HA at Day +3	80	0.0008	-0.002	0.004	0.59
ST2 at Day +3	80	0.010	-0.018	0.038	0.48

Table S15. Association between biomarker levels and idiopathic pneumonia syndrome (IPS)

Biomarker and Time Point	n	Beta estimate	95% CI		p-value
L-Ficolin at Day +3	72	0.00056	0.00005	0.0010	0.032*
HA at Day +3	72	-0.0002	-0.006	0.0053	0.94
ST2 at Day +3	72	0.0014	-0.042	0.045	0.95

Note*: p-value < 0.05.

Table S16. Association between biomarker levels and overall graft-versus-host disease (GVHD) severity

Biomarker and Time Point	n	Beta estimate	95% CI		p-value
L-Ficolin at Day +3	72	-0.0003	-0.0006	-0.00008	0.011*
HA at Day +3	72	-0.0001	-0.002	0.0019	0.92
ST2 at Day +3	72	0.0012	-0.019	0.021	0.90

Note*: p-value < 0.05.

Table S17. Association between IL-6 and TNFR1 levels and overall graft-versus-host disease (GVHD) severity

Biomarker and Time Point	n	Beta estimate	95% CI		p-value
IL-6 at Day +3	72	0.0006	-0.0032	0.0044	0.77
TNFR1 at Day +3	72	0.00009	-0.0004	0.0005	0.70

Table S18. Association between biomarker levels and infections

Biomarker and Time Point	n	Beta estimate		95% CI		p-value
L-Ficolin at Day +3	18					0.44
		Bacterial and Viral vs. Bacterial	-0.0004	-0.0020	0.0013	0.65
		Virial vs. Bacterial	0.0003	-0.0002	0.0009	0.25
HA at Day +3	18					0.50
		Bacterial and Viral vs. Bacterial	0.0012	-0.0028	0.0051	0.57
		Virial vs. Bacterial	-0.0023	-0.0070	0.0024	0.33
ST2 at Day +3	18					0.22
		Bacterial and Viral vs. Bacterial	0.0059	-0.0268	0.0385	0.73
		Virial vs. Bacterial	-0.0955	-0.2066	0.0156	0.09

Table S19. Descriptive statistics of biomarkers in subcategorized age in patients without SOS.

	Age in patients without SOS, mean ± Standard Deviation					
**Biomarker**	**Overall N=70**	**0 to < 3 N=11**	**>= 3 to < 10 N=20**	**>= 10 to < 16 N=25**	**>= 16 to <= 22 N=14**	**P-value**
**L-Ficolin Day +3**	3722 ± 2162	3039 ± 1909	3525 ± 1846	3864 ± 1648	4285 ± 3344	0.52
**HA Day +3**	167 ± 236	203 ± 193	257 ± 354	124 ± 175	88 ± 51	0.13
**ST2 Day +3**	26 ± 18	21 ± 20	24 ± 19	26 ± 19	32 ± 12	0.47

Note: P-value is based on ANOVA.

Figure S1. Kaplan-Meier estimates of overall survival (OS) on day 100 post-transplant

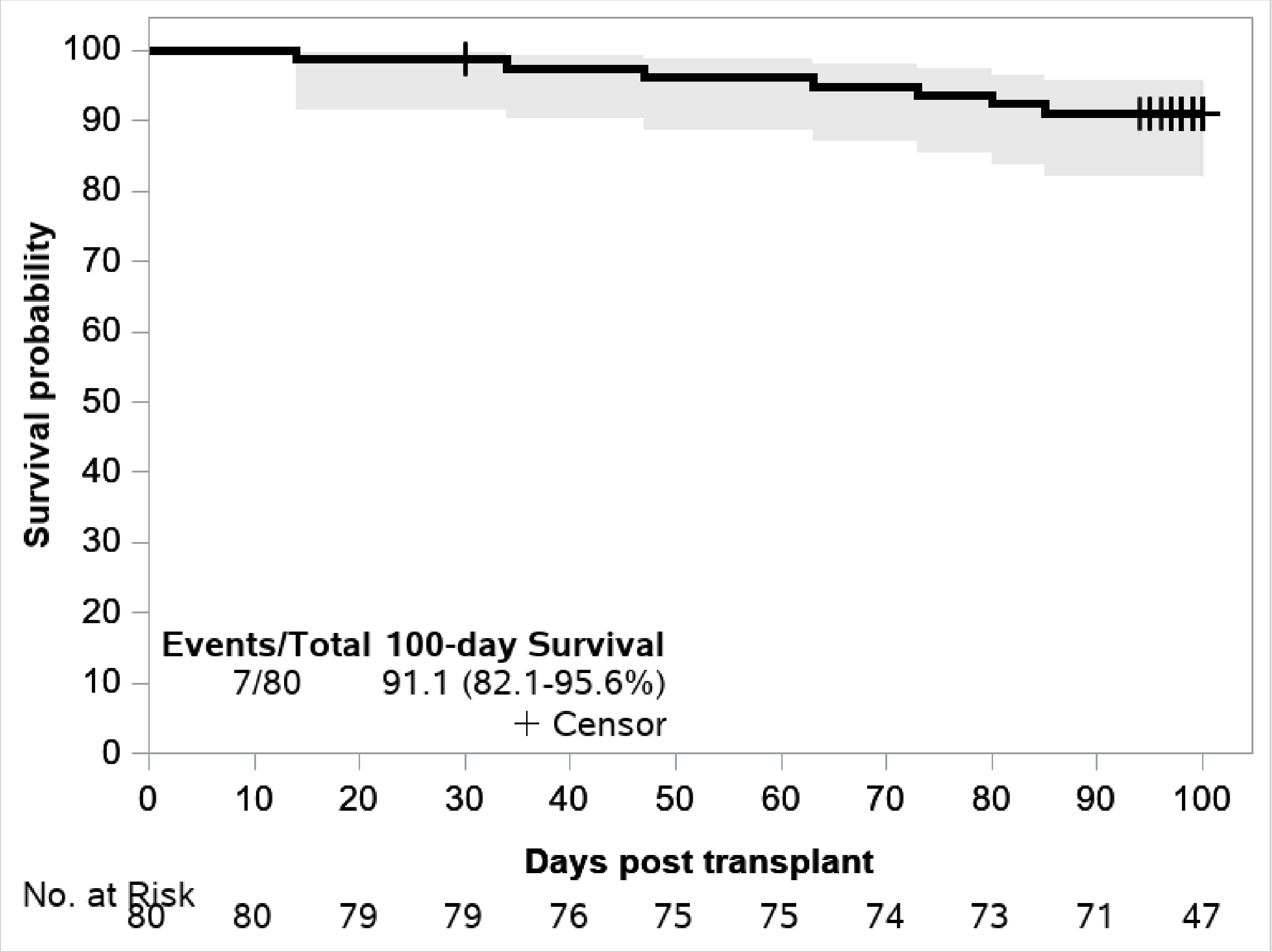

Figure S2A. ROC curves for day 3 biomarkers

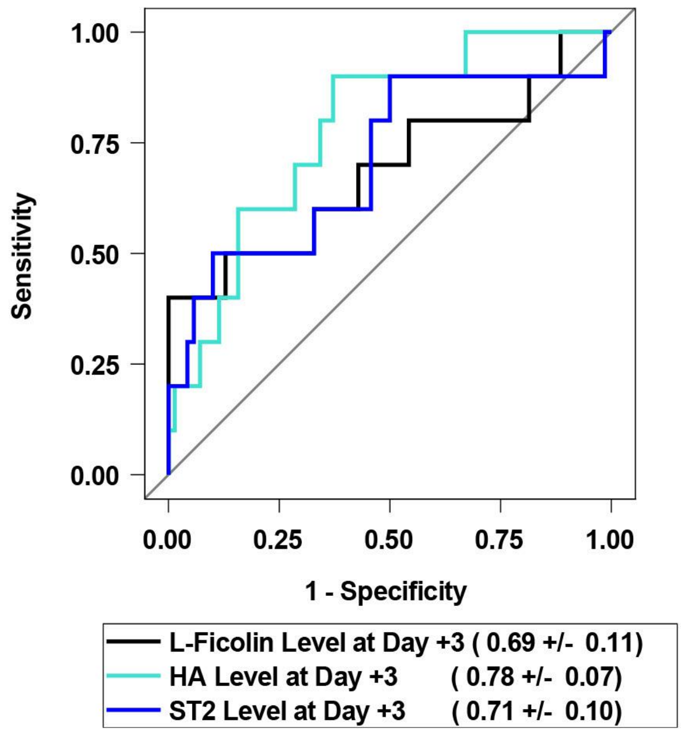

Figure S2B. ROC curves for day 7 biomarkers

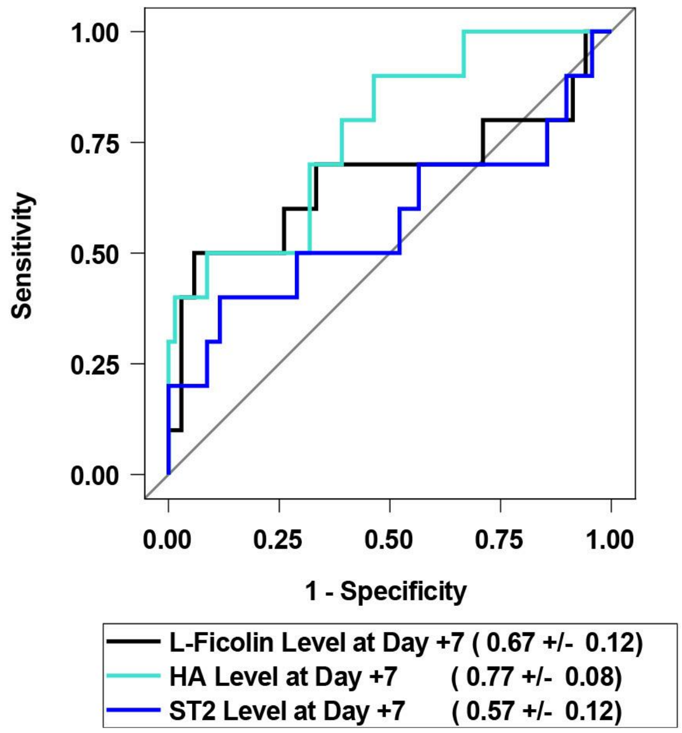

Figure S3. Proposed preemptive trial design

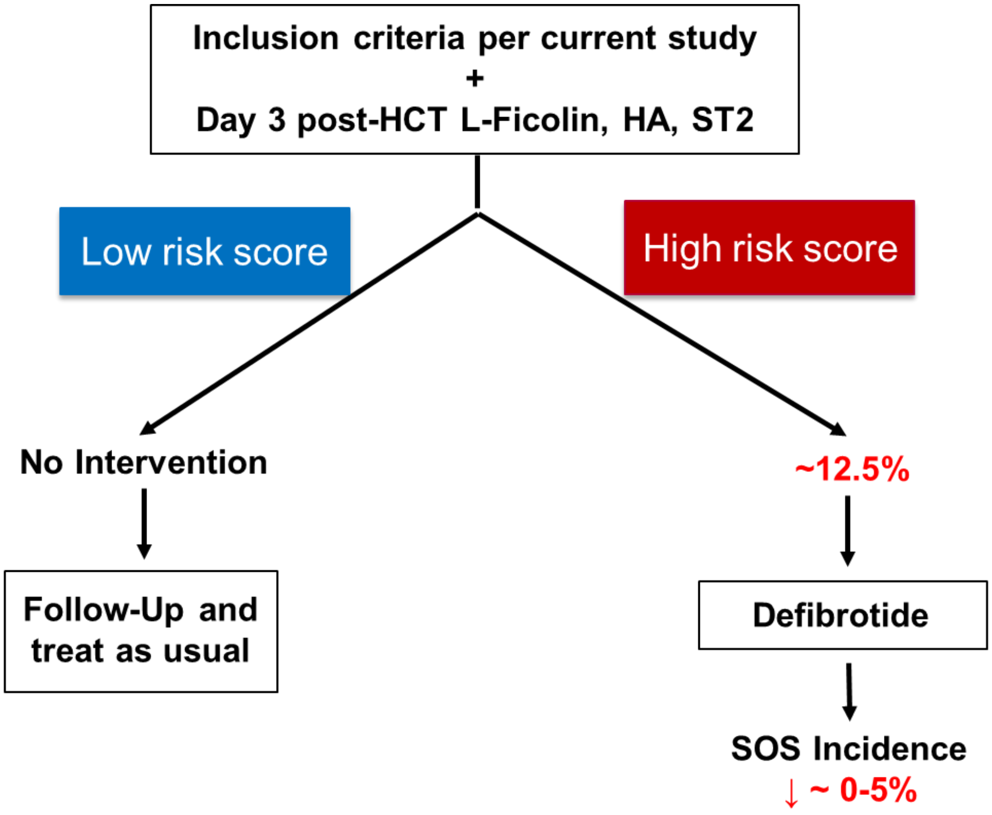

Supplement: Supplemental data [file jciinsight-8-168221-s129.pdf]
